# Non-neutralizing anti-type I interferon autoantibodies could increase thrombotic risk in critical COVID-19 patients

**DOI:** 10.3389/fimmu.2025.1556731

**Published:** 2025-03-17

**Authors:** Mario Framil, Lydia García-Serrano, Francisco Morandeira, Juan Francisco Luchoro, Arnau Antolí, Jose Luis Gomez-Vazquez, Àngels Sierra-Fortuny, Xavier Solanich

**Affiliations:** ^1^ Immunology Department, Centre Diagnòstic Biomèdic, Hospital Clínic de Barcelona, Barcelona, Spain; ^2^ Immunology Department, Hospital Universitari de Bellvitge, L’Hospitalet de Llobregat, Spain; ^3^ Bellvitge Biomedical Research Institute (IDIBELL), L’Hospitalet de Llobregat, Spain; ^4^ Internal Medicine Department, Hospital Universitari de Bellvitge, L’Hospitalet de Llobregat, Spain

**Keywords:** non-neutralizing autoantibodies, type I interferon, COVID-19, thrombotic complications, endothelial dysfunction, ICU patients

## Abstract

During the COVID-19 pandemic, approximately 15% of patients with severe COVID-19 pneumonia were reported to have neutralizing anti-type I interferon (IFN) autoantibodies, which impaired the antiviral response and led to a poorer prognosis. However, the physiological impact of non-neutralizing autoantibodies remains unclear. In our cohort of COVID-19 patients admitted to intensive care, the presence of non-neutralizing anti-type I IFN autoantibodies increased the risk of thrombotic complications, likely via a cytokine carrier mechanism, prolonging the half-life of cytokines and dysregulating vascular endothelial function. Previous studies have associated non-neutralizing anti-type I IFN autoantibodies with an increased risk of cardiovascular complications in autoimmune diseases like systemic lupus erythematosus, but their relevance in infectious diseases remains uncertain. Stratifying anti-type I IFN autoantibodies based on their neutralizing capacity may have clinical significance not only in terms of susceptibility to infectious diseases but also in predicting cardiovascular and thrombotic events.

## Introduction

The COVID-19 pandemic heightened interest in anti-cytokine autoantibodies, particularly anti-type I IFN autoantibodies. Bastard et al. (1) revealed high titers of neutralizing autoantibodies against type I IFNs in up to 10% of severe COVID-19 patients. These autoantibodies compromised antiviral responses and increased the risk of severe COVID-19, especially in males over 65 years old, with a prevalence of approximately 20% in patients who died (2). Subsequent global studies confirmed these findings, emphasizing the role of neutralizing autoantibodies against type I IFNs as a significant risk factor for severe COVID-19. Anti-IFN neutralizing autoantibodies have since been linked to severe outcomes in other diseases, including severe influenza pneumonia, neurological complications associated with West Nile virus, and adverse responses to the yellow fever vaccine (2).

Most studies have focused on how neutralizing autoantibodies impair IFN signaling. However, non-neutralizing autoantibodies, which make up over 50% of detected antibodies (2, 3), have also been observed to play physiological roles. One of these roles is the stabilization of cytokines, such as IL-6, by preventing their degradation and extending their half-lives *in vivo*, likely through the formation of cytokine-antibody complexes (4). These complexes may subsequently recycle into the bloodstream, contributing to a dynamic equilibrium between free cytokines and those bound to antibodies. This recycling process is mediated by neonatal Fc receptors, predominantly expressed on vascular endothelial and myeloid cells, thereby ensuring the sustained presence of cytokines in circulation (5).

A similar process has been observed in systemic autoimmune diseases, such as systemic lupus erythematosus (SLE), where anti-type I IFN autoantibodies exhibit distinct roles based on their neutralizing capacity (6). Neutralizing anti-cytokine autoantibodies have been associated with decreased interferon-pathway activity and reduced disease activity in SLE patients. In contrast, non-neutralizing autoantibodies, particularly IFN-α2, have been linked to elevated cytokine concentrations in peripheral blood, which correlates with an increased risk of cardiovascular and respiratory complications (7). Given these observations, we aimed to evaluate the clinical outcomes in life-threatening COVID-19 patients based on the presence of non-neutralizing type I IFN autoantibodies.

## Materials and methods

Study Design and Patients: This retrospective study involved COVID-19 patients admitted to the Intensive Care Unit (ICU) of Hospital Universitari de Bellvitge from March 2020 to March 2021, with confirmed SARS-CoV-2 infection. Data were obtained from routine clinical practice and anonymized. Personal and clinical information was collected in compliance with the Spanish Data Protection Act (Ley Orgánica 3/2018, December 5, on Personal Data Protection). Informed consent was waived due to the study’s retrospective nature and the mandatory isolation measures during in-hospital care. The protocol was approved by the Ethics Committee of Hospital Universitari de Bellvitge (Barcelona, Spain; approval number PR40/21).

Clinical and Laboratory Variables: Demographic, clinical, and evolutive data during hospitalization were recorded, and laboratory variables were collected upon ICU admission. Detailed descriptions of these clinical variables can be found in the original study by Solanich et al. (3).

Autoantibodies Against Type I IFNs: Autoantibodies against IFN-α2 and IFN-ω were analyzed using an ELISA technique as described in Solanich et al. (3). In brief, NUNC MaxiSorp™ 96-well ELISA plates (Thermo Fisher Scientific) were coated with recombinant human IFN-α2 or IFN-ω (1 mg/L in 100 μL coating buffer) and incubated overnight at 4°C. After three PBS washes, plates were blocked with PBS-5% nonfat milk for 1 h at room temperature with continuous shaking at 600 rpm, washed with PBS-Tween 0.005%, and incubated with 100 μL of 1:50 diluted serum samples (patients/controls) in HPE buffer (Sanquin) for 2 h with shaking at room temperature. After washing, Fc-specific HRP-conjugated goat anti-human IgG (Nordic-MUbio, 2 mg/L) was added and incubated for 1 h. Following washes, TMB substrate was added for 10 min, and the reaction was stopped with 0.18 M H_2_SO_4_. Optical density at 450 nm was measured. Samples were considered positive if exceeding the mean value plus two standard deviations of a non-COVID-19 control group (3).

Neutralizing Autoantibodies Against Type I IFNs: The neutralizing ability of these autoantibodies at high concentrations of IFN-α2 and IFN-ω (10 ng/mL) was assessed using a functional *in vitro* Dual-Luciferase reporter assay, as described in Solanich et al. (3). In brief, HEK293T cells were transfected with firefly luciferase plasmids under human ISRE promoters (pGL4.45) and a constitutively expressing Renilla luciferase plasmid (pRL-SV40) for normalization. Transfection was performed with X-tremeGene 9 (Millipore-Sigma) for 36 h. Cells were then incubated in Dulbecco’s modified Eagle medium (DMEM, Thermo Fisher Scientific) with 10% healthy control or patient serum/plasma, either unstimulated or stimulated with IFN-α2 or IFN-ω (10 ng/mL) for 16 h at 37°C. Each sample was tested once. Luciferase levels were measured using Dual-Glo reagent (Promega Corp., Madison, WI, USA), according to the manufacturer’s protocol. Firefly luciferase values were normalized against Renilla luciferase values (3).

Statistical Analysis: Data are presented as median with interquartile range (IQR) for continuous variables, and as frequency rates and percentages for categorical variables. Comparisons for continuous variables were made using the Mann-Whitney U test, while comparisons for categorical variables were assessed using the chi-square test or Fisher’s exact test, as appropriate. Odds Ratios (OR) with 95% confidence intervals (CI) were calculated using logistic regression models or contingency tables, depending on the variable distribution. All tests were conducted with 95% confidence intervals and a significance level of 5%.

## Results

Serum samples from 275 patients were tested for autoantibodies against type I IFNs (IFN-α2 and IFN-ω) using ELISA. Autoantibodies were detected in 49 patients (17.8%). Among them, 23 (46.9%) had non-neutralizing autoantibodies. Within this group, 5 patients (21.7%) were positive for IFN-α2, 7 (30.4%) for IFN-ω, and 11 (47.8%) for both interferons. Male gender was more prevalent among patients with non-neutralizing autoantibodies compared to those without them (91.3% vs. 75.4%; p = 0.010). No significant difference in median age was found between groups (61 years, IQR 55–67 vs. 64 years, IQR 57–71; p = 0.112) (**Table 1**).

**Table 1:** Main demographics and clinical complications of severe COVID-19 patients with non-neutralizing anti-type I IFN autoantibodies compared to patients without anti-type I IFN autoantibodies.

Variable	Patients with nnAAb anti-IFN I (n= 23)	Patients without AAb anti-IFN I (n=226)	*p*-value	OR (95% CI)
Demographics				
Age, yr; median (IQR)	61 (55-67)	64 (57-71)	0.112	–
Sex (male); n (%)	21 (91,3%)	166 (75,4%)	**0.010**	–
Complications; n (%)				
Exitus	13 (56.52%)	118 (52.21%)	0.827	1.12 (0.49–2.56)
Multiorganic failure	6 (26.09%)	45 (19.91%)	0.587	1.42 (0.51–3.98)
Neurological complications	7 (30.43%)	65 (28.76%)	0.814	1.09 (0.45–2.64)
Thrombotic complications	12 (52.17%)	33 (14.60%)	**<0.001**	**4.05** (1.64–9.97)
Hemorrhagic complications	1 (4.35%)	22 (9.73%)	0.705	0.42 (0.06–3.22)
Cardiovascular complications	8 (34.78%)	43 (19.03%)	0.100	2.22 (0.87–5.67)
Cardiorespiratory arrest	3 (13.04%)	24 (10.62%)	0.724	1.26 (0.35–4.52)
Infection	17 (73.91%)	171 (75.66%)	0.804	0.91 (0.35–2.38)
Sepsis	11 (47.83%)	112 (49.56%)	1	0.94 (0.43–2.06)
Septic shock	5 (21.74%)	61 (26.99%)	0.805	0.75 (0.27–2.11)
Digestive complications	5 (21.74%)	30 (13.27%)	0.339	1.81 (0.62–5.28)
Airway complications	4 (17.39%)	25 (11.06%)	0.321	1.70 (0.54–5.40)

The comparison excludes patients with neutralizing autoantibodies. For further details on the analysis of neutralizing autoantibodies, see Solanich et al. (3).

Bold values indicate statistical significance.

**Figure 1:**
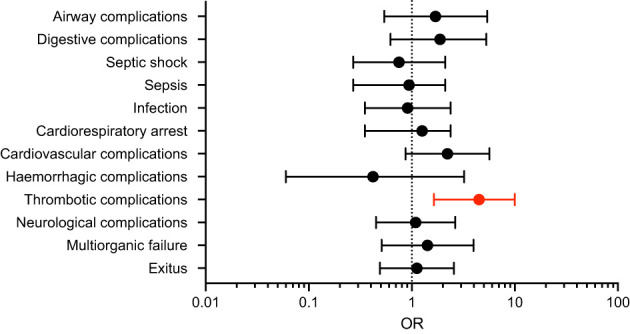
Odds of complications in severe COVID-19 patients with non-neutralizing anti-type I IFN autoantibodies. Forest plot showing the Odds Ratios (OR) with 95% confidence intervals (CI) for different complications. The dotted line represents an OR of 1 (no association). Thrombotic complications show significantly higher odds (highlighted in red). Error bars represent confidence intervals.

Interestingly, the presence of non-neutralizing autoantibodies against type I IFNs was significantly associated with thrombotic complications compared to their absence (12 [52.2%] vs. 33 [14.6%]; p < 0.001) (**Table 1**), having four times higher odds of developing thrombotic complications (OR 4.054 [95% CI 1.638-9.972]) (**Figure 1**). Additionally, there was also a higher tendency of cardiovascular complications (8 [34.8%] vs. 43 [18.8%]; p = 0.098), especially among fatal cases (6 [46.2%] vs. 26 [21.7%]; p = 0.081). No other clinical complications did reach statistical significance.

## Discussion

Our study underscores the significant association between non-neutralizing autoantibodies against type I interferons and an increased risk of thrombotic complications in critically ill COVID-19 patients. The role of non-neutralizing autoantibodies remains unclear, particularly regarding their physiological significance or potential impact on disease progression.

Previous studies have reported the presence of anti-type I interferon autoantibodies in approximately 10% of patients with SLE and primary Sjögren’s syndrome (6, 7). In these cohorts, patients with neutralizing autoantibodies experienced better clinical outcomes, showing less severity and fewer relapses compared to those without autoantibodies. Notably, patients with non-neutralizing antibodies exhibited higher levels of IFN-α2 than those without, presenting clinical symptoms as severe or even more pronounced. Furthermore, this group showed a higher percentage of cardiovascular and respiratory complications.

Moreover, type I interferons play a crucial role in vascular endothelial homeostasis by maintaining a balance between procoagulant and fibrinolytic factors. Sustained elevation of type I IFN levels in the vascular endothelium can disrupt this balance, leading to a hypercoagulable state (8, 9). This dysregulation has been linked to endothelial dysfunction and an increased risk of thrombosis, with prolonged exposure to type I interferons contributing to microangiopathy and impaired endothelial cell functions (8,^9^). In our cohort, the presence of non-neutralizing autoantibodies was significantly associated with a high incidence of thrombotic complications. This may be due to non-neutralizing autoantibodies extending cytokine half-life in circulation, further disrupting endothelial homeostasis.

Understanding the mechanisms underlying the association between non-neutralizing autoantibodies and thrombotic complications could significantly enhance outcomes in high-risk patients, not only in COVID-19 but also in other infectious diseases driven by anti-type I interferon autoantibodies, as well as in systemic autoimmune diseases. Further research is needed to validate these findings and explore whether similar associations are observed in other conditions, helping to clarify the role of non-neutralizing autoantibodies in disease progression and their potential as therapeutic targets.

## Data Availability

The original contributions presented in the study are included in the article/supplementary material. Further inquiries can be directed to the corresponding author.
